# Imported Cutaneous Leishmaniasis from Peru Caused by *Leishmania (Viannia) guyanensis* in a Brazilian Patient: Case Report and In Vitro Drug Susceptibility Analysis

**DOI:** 10.3390/tropicalmed9110264

**Published:** 2024-11-05

**Authors:** Elizabeth M. Coser, Juliana I. Aoki, Cristiele Saborito, Stephane de la Roca, João Paulo T. Brufatto, Rodrigo Angerami, Rafael F. Stelini, Paulo Eduardo N. F. Velho, Adriano C. Coelho

**Affiliations:** 1 Departamento de Biologia Animal, Instituto de Biologia, Universidade Estadual de Campinas (UNICAMP), Campinas 13083-862, Brazil; elizabethmcoser@hotmail.com (E.M.C.); juaoki@gmail.com (J.I.A.);; 2 Departamento de Clínica Médica, Faculdade de Ciências Médicas, Universidade Estadual de Campinas (UNICAMP), Campinas 13083-894, Brazilangerami@unicamp.br (R.A.); pvelho@unicamp.br (P.E.N.F.V.); 3 Departamento de Patologia, Faculdade de Ciências Médicas, Universidade Estadual de Campinas (UNICAMP), Campinas 13083-894, Brazil

**Keywords:** *Leishmania guyanensis*, cutaneous leishmaniasis, pentavalent antimony, treatment failure

## Abstract

In South America, cutaneous leishmaniasis is caused by several species of the parasite of the genus *Leishmania*. Here, we describe an imported case of cutaneous leishmaniasis acquired in Peru by a Brazilian patient during her travel to Iquitos. Infection by *Leishmania* parasites was confirmed by histopathologic examination, and the patient was treated with pentavalent antimony (Pentostam), without clinical response. Molecular typing was performed by sequencing the ribosomal DNA internal transcribed spacer and *heat-shock protein 70* gene, which identified the parasites as *Leishmania guyanensis*. The clinical isolate was similarly susceptible to amphotericin B, pentamidine, and miltefosine as the reference strain, while for pentavalent antimony, this clinical isolate was more susceptible than the reference strain, even though its susceptibility in vitro was still considered low. The patient was then treated with liposomal amphotericin B, with clinical improvement of the lesions.

## 1. Introduction

Leishmaniasis is a complex of vector-borne diseases transmitted through bites from infected female phlebotomine sandflies and caused by the parasite of the genus *Leishmania.* Cutaneous leishmaniasis (CL) is generally characterized by an ulcer at the site of the sandfly’s bite that can take several months to heal. It may progress to more severe manifestations in approximately 10% of cases and can be classified as disseminated, diffuse cutaneous, or mucocutaneous leishmaniasis, depending on the parasite species and host immune response [1]. In Peru, a highly endemic country in South America, six species are responsible for CL: *Leishmania (Viannia) peruviana,* which is the most prevalent species, and *L. (V.) braziliensis, L. (V.) guyanensis*, *L. (V.) lainsoni*, *L. (V.) shawi,* and *L. (Leishmania) amazonensis* [2,3,4]. In 2022, 37,890 new cases of CL were reported in Latin America, and 5756 (or 15.2%) of these cases occurred in Peru. Almost 50% of the Peruvian population live in areas with the transmission of disease, and 37% of cases in Peru occur at international borders [5]. Among the main risk factors associated with disease transmission are poor-quality of houses and living close to forested areas, and cases are often related to traveling, migration, and military conflicts [1,6,7].

The treatment of leishmaniasis is based on a limited number of drugs that include pentavalent antimonials (SbV) in their two formulations, sodium stibogluconate (Pentostam) and meglumine antimoniate (Glucantime). These drugs inhibit trypanothione reductase, a mitochondrial enzyme, increasing the sensibility of the parasite to the oxidative stress generated by macrophages, and also inhibit glycolytic enzymes and fatty acid oxidation [8]. The mechanism of resistance is related to the diminished ability of intracellular amastigotes to reduce SbV to trivalent antimony (SbIII) [9]. Other mechanisms involve drug sequestration and/or efflux and an increase in intracellular thiol levels as a defense mechanism to combat the oxidative stress generated by SbV [8]. In addition, other drugs include amphotericin B (AmB), a second-line drug that also has two formulations, deoxycholate and the less toxic formulation, liposomal AmB (L-AmB); and pentamidine (PEN), the drug least often used, owing to the high frequency of side effects [10]. In South America, SbV is still considered the first-line treatment for CL, with significant variation in cure rates, from 26.3 to 80% [4,10,11], while both AmB formulations have cure rates higher than 80% [10]. Miltefosine (MF) is the only oral drug available for CL treatment in South America with cure rates higher than 70% [10]. Additionally, the presence of *Leishmania* RNA virus 1 (LRV1), which resides as an endosymbiont in parasites of the subgenus *Viannia,* has already been associated with treatment failure in patients treated with SbV and PEN [12,13].

## 2. Case Presentation

**Figure 1:**
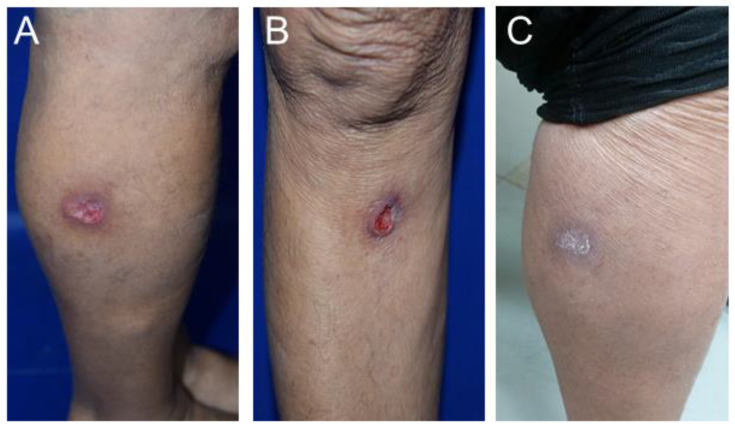
Delimited ulcerated skin lesions on the inferior right leg caused by *L. (V.) guyanensis* and acquired by the patient during her travel to Peru. Lesions on the right leg before treatment (**A**,**B**) and one of the lesions after treatment with L-AmB (**C**).

**Figure 2:**
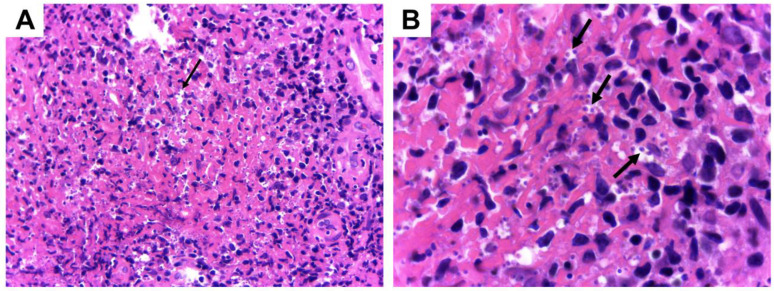
Histological analysis of a skin biopsy showing amastigotes of *Leishmania* parasites in tissue macrophages (arrows). Slides were stained with Giemsa, and the images obtained are at a magnification of ×400 and ×1000 ((**A**,**B**)**,** respectively).

We describe the case of a patient that acquired CL during her travel to the city of Iquitos, located in the Amazon region of Peru. The patient, a 74-year-old Brazilian woman from Valinhos city, went to a hospital in Peru in June of 2022 with two delimited ulcerated lesions on her right leg. After parasitological diagnostic testing by press–imprint–smear, the presence of intracellular *Leishmania* amastigotes was confirmed. The patient was treated with sodium stibogluconate (Pentostam) for 7 days (300 mg/day), with no clinical response. Back in Brazil, the patient sought medical attention at the Hospital de Clínicas of UNICAMP, presenting with two ulcerated lesions on her leg measuring 5 × 4 cm and 7 × 4 cm (Figure 1A,B). A skin biopsy of one of these lesions was submitted for histopathological examination, which confirmed the presence of *Leishmania* amastigotes (Figure 2) and was subsequently used for parasite isolation. Promastigotes of the clinical isolate IMLC (MHOM/PE/2022/IMLC) were successfully obtained in supplemented 199 medium after approximately 10 days at 25 °C, as previously described [14]. The species identification of the isolate was performed through PCR amplification followed by the Sanger sequencing of the internal transcribed spacer (ITS) DNA and the *heat-shock protein 70 (hsp70)* gene [15,16]. The full ITS nucleotide sequence of the IMLC isolate displayed more than 98% identity with the ITS of other *L. (V.) guyanensis* strains and isolates deposited in GenBank and 98.57% identity with the *L. (V.) guyanensis* M4147 strain (MHOM/BR/75/M4147), considered a reference by the WHO [17]. For the *hsp70* gene, the nucleotide sequence of the IMLC isolate showed 99.77% identity with that of the M4147 reference strain and other *L. (V.) guyanensis* strains from Peru and French Guiana. The GenBank accession numbers of the ITS and *hsp*70 sequences of the IMLC isolate are PP397097 and PP405571, respectively.

To evaluate whether the failure of treatment in the patient could be due to SbV resistance in the IMLC isolate, we tested the in vitro susceptibility of both the amastigote and promastigote forms of the parasite to SbV and also to other antileishmanial drugs to which the patient had not been previously exposed (AmB deoxycholate, MF and PEN). Log-phase promastigotes (2 × 10^6^ per well) were incubated for 24 h in the presence of serially diluted drugs [14] then submitted for MTT (3-(4,5-dimethylthiazol-2yl)-2,5-diphenyl tetrazoline bromide) colorimetric assay. All 50% effective concentration (EC_50_) values were determined using the M4147 strain as a control. The IMLC isolate in the promastigote form was at least 2-fold more susceptible to SbIII, AmB, and MF than the M4147 strain (Table 1). For PEN, the EC_50_ of the IMLC isolate was around 2-fold less susceptible (Table 1).

For parasites in the intracellular amastigote stage, we first determined the infection rate and the average number of amastigotes per macrophage of the IMLC isolate and reference strain. Bone-marrow-derived macrophages (BMDMs) were infected with stationary-phase promastigotes in a 30:1 ratio (parasites/macrophage) and incubated at 34 °C in a 5% CO_2_ atmosphere for 3–4 h. The IMLC isolate had a rate of infection of 84 ± 6.72%, while the infection rate of the M4147 strain was 60 ± 6.85%. The number of amastigotes per macrophage was 6.09 ± 1.85 and 5.86 ± 0.66 for the IMLC isolate and M4147 strain, respectively. Following BMDM infection, non-internalized parasites were removed, and the drugs were added as follows: AmB (150 to 0.78 nM), PEN (0.4 to 0.01 µM), and MF (20 to 0.31 µM) for 72 h, and SbV (1000 to 25 µM) for 144 h, as previously described [14]. Although the patient had not responded to SbV treatment (Pentostam), the IMLC isolate was more susceptible to SbV (Glucantime) than the M4147 strain, and its EC_50_ value was around 5-fold lower (>1000 versus 221.8 µM) (Table 1), even though its susceptibility is still considered low compared to values reported in other studies [18]. In fact, the M4147 strain was intrinsically resistant to SbV in vitro, and we were not able to determine its EC_50_ value (Table 1). This strain was obtained from a human host before any clinical treatment with SbV or any other antileishmanial drug [17]. *L. (V.) guyanensis* isolates from patients from Peru, obtained after cure with SbV, were found to be either susceptible or resistant to this drug (with EC_50_ values below 40 µM and above 220 µM, respectively) [18]. Furthermore, twenty-nine isolates from Colombian patients typed as *L. (V.) guyanensis* were susceptible to SbV in vitro, and none were classified as resistant to the drug (EC_50_ values >90 µM) [19]. Finally, the cure rates in patients infected by *L. (V.) guyanensis* and treated with SbV range from 75.6 to 91.7% in Peru [3,4]. These findings indicate no clear association between in vitro SbV susceptibility and CL treatment outcome, although the low susceptibility to SbV of the IMLC isolate may have contributed to the patient’s treatment failure. It is important to state that the patient was exposed to a single course of SbV treatment, suggesting an intrinsic resistance to the SbV (or primary resistance) of this isolate and supporting the plausibility of anthroponotic transmission for this patient [20].

The EC_50_ values were similar between the IMLC isolate and the M4147 strain for AmB and MF, and both were considered susceptible (Table 1). For PEN, the EC_50_ values were higher than the value for BMDMs (0.36 ± 0.02 µM) [14] and thus could not be determined (Table 1). Despite this low susceptibility in vitro, PEN has been used for the treatment of CL caused by *L. (V.) guyanensis,* with efficacy rates of up to 100% in patients from Brazil [21]. We also investigated the IMLC isolate for the presence of LRV1, which has previously been associated with treatment failure in human infections due to *L. (V.) guyanensis* and *L. (V.) braziliensis* [12,13]. However, it is important to state that there is still no consensus in the literature regarding the correlation between treatment failure and the presence of LRV1 in *Leishmania* parasites [12,13,22,23]. For example, in infections with *L. (V.) guyanensis,* treatment failure rates reached of 40% in patients treated with PEN, and no correlation with the presence of LRV1 was observed [22]. In the IMLC isolate, LRV1 was not detected by RT-PCR, while the expected PCR product was amplified in the M4147 strain, previously described as a host for LRV1 [24] (Supplementary Materials, Figure S1). Finally, these findings indicate that treatment failure may be due to factors unrelated to LRV1 or drug resistance of the parasite, such as the causative *Leishmania* species, disease duration and presence of concomitant-distant lesions, patient age, and genetic polymorphisms related to the immune response [4,25,26]. Regarding *Leishmania (Viannia)* species, it has already been demonstrated that the causative species may affect treatment outcome in patients treated with SbV [3,20], indicating the importance of species identification in guiding the treatment of CL. Due to the absence of a clinical response to SbV treatment, the patient was hospitalized and treated with intravenous L-AmB (200 mg/day) for 14 days, with clinical improvement in the lesions (Figure 1C). One year after clinical cure, the patient continues to be monitored at Hospital de Clínicas of UNICAMP, and no relapse has been reported so far.

## 3. Conclusions

In summary, we describe an imported case of CL caused by *L. (V.) guyanensis* acquired by a Brazilian patient during her travel to Peru. Despite treatment failure after SbV therapy, no clear correlation with in vitro SbV susceptibility was found for this isolate. On the other hand, the clinical response to the treatment with L-AmB corroborated the in vitro susceptibility findings, demonstrating that the IMLC isolate is susceptible to amphotericin B.

## Figures and Tables

**Table 1:** In vitro susceptibility of promastigotes and intracellular amastigotes of the *L. (V.) guyanensis* M4147 strain and the IMLC isolate to antileishmanial drugs.

Drugs	Promastigotes		Intracellular Amastigotes		CC_50_ ^c^
	M4147	IMLC	M4147	IMLC	
SbIII ^a^	20.58 ± 2.88	10.82 ± 4.25	-	-	-
SbV ^a^	-	-	>1000	221.8 ± 18	>2000
AmB ^b^	31.73 ± 2.81	15.26 ± 2.43	13.11 ± 1.11	9.8 ± 2.95	127.36 ± 0.94
PEN ^a^	0.41 ± 0.078	0.91 ± 0.07	>0.4	>0.4	0.36 ± 0.02
MF ^a^	46.4 ± 4.37	21.52 ± 5.43	13.5 ± 4.52	9.05 ± 2.3	49.52 ± 2.93

^a^ EC_50_ mean values ± standard deviation in µM of three independent experiments. ^b^ EC_50_ mean values ± standard deviation in nM of three independent experiments. ^c^ The CC_50_ values for SbV, AmB, PEN, and MF against BMDMs were previously determined [14]. (-) Data not determined.

## Data Availability

All data presented in this study are available upon request. The ITS and *hsp*70 sequences obtained in this study have been deposited in GenBank under accession numbers PP397097 and PP405571, respectively.

## Supplementary Materials

The following supporting information can be downloaded at: https://www.mdpi.com/article/10.3390/tropicalmed9110264/s1, Figure S1: Investigation of LRV1 in the IMLC isolate, as previously described [27].

## Abbreviations

AmB, amphotericin B; BMDMs, bone marrow-derived macrophages; CC_50_, 50% cytotoxicity concentration; CL, cutaneous leishmaniasis; EC_50_, 50% effective concentration; ITS, internal transcribed spacer; LRV1, *Leishmania* RNA virus 1; MF, miltefosine; PEN, pentamidine; SbIII, trivalent antimony; SbV, pentavalent antimony.
