# Supplementary material for: Imported Cutaneous Leishmaniasis from Peru Caused by Leishmania (Viannia) guyanensis in a Brazilian Patient: Case Report and In Vitro Drug Susceptibility Analysis
Source: Trop Med Infect Dis. 2024 Nov 5;9(11):264. doi: 10.3390/tropicalmed9110264 (PMC11598126; doi:10.3390/tropicalmed9110264)
Supplement: Supplementary file 1 [file tropicalmed-09-00264-s001.zip › tropicalmed-3260712-supplementary.pdf]

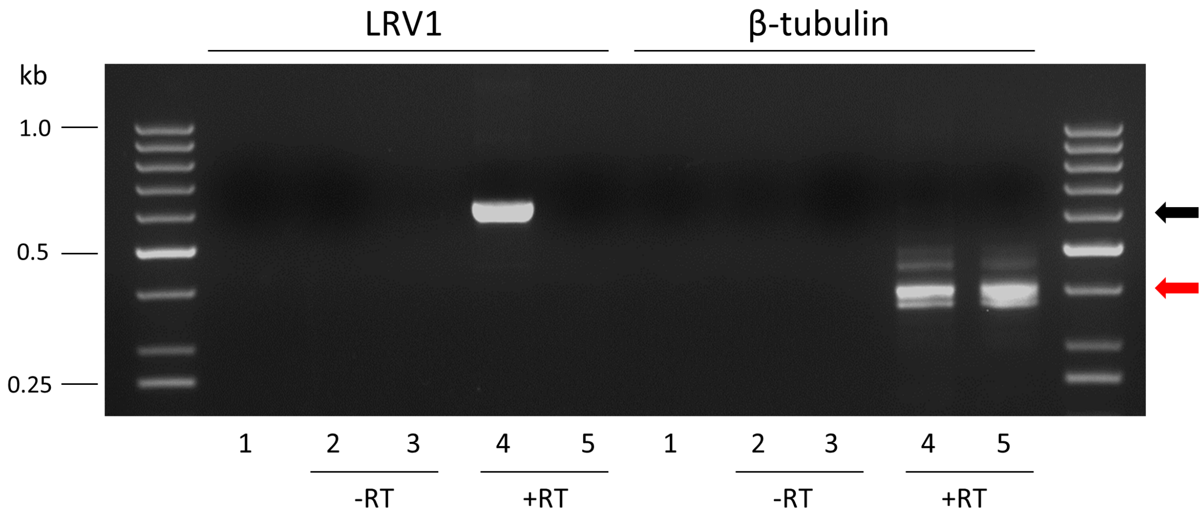

Figure S1. Investigation for LRV1 in the IMLC isolate. To detect LRV1, a RT-PCR reaction that targets LRV1 capsid mRNA amplifying a 688 bp fragment (black arrow) was performed as previously described [1]. The *L. (V.) guyanensis* M4147 strain is positive for LRV1 and was used as a control. The β-tubulin gene transcript whose product has approximately 400 bp (red arrow) was used as a control for the presence of cDNA in samples. Amplified products were analyzed in an agarose gel electrophoresis stained with ethidium bromide. Legend: 1, absence of cDNA; 2 and 4, *L. (V.) guyanensis* M4147 strain (positive control for LRV1); 3 and 5, IMLC isolate. Presence or absence of reverse transcriptase in the cDNA synthesis reaction are represented by + RT and - RT respectively.

Reference

1. Ferreira, B.A.; Coser, E.M.; Saborito, C.; Yamashiro-Kanashiro, E.H.; Lindoso, J.A.L.; Coelho, A.C. In vitro miltefosine and amphotericin B susceptibility of strains and clinical isolates of Leishmania species endemic in Brazil that cause tegumentary leishmaniasis. *Exp Parasitol* **2023**, *246*, 108462, doi:10.1016/j.exppara.2023.108462.
